# Non-Linear Correlation Between Tumor Size and Survival Outcomes for Parathyroid Carcinoma: A SEER Population-Based Cohort Study

**DOI:** 10.3389/fendo.2022.882579

**Published:** 2022-07-01

**Authors:** Kun Zhang, Anping Su, Xiaofei Wang, Wanjun Zhao, Linye He, Tao Wei, Zhihui Li, Jingqiang Zhu, Ya-Wen Chen

**Affiliations:** ^1^ Thyroid and Parathyroid Surgery Center, Sichuan University West China Hospital, Chengdu, China; ^2^ Department of Otolaryngology, Icahn School of Medicine at Mount Sinai, New York, NY, United States; ^3^ Department of Cell, Developmental and Regenerative Biology, Icahn School of Medicine at Mount Sinai, New York, NY, United States; ^4^ Black Family Stem Cell Institute, Icahn School of Medicine at Mount Sinai, New York, NY, United States; ^5^ Institute for Airway Sciences, Icahn School of Medicine at Mount Sinai, New York, NY, United States

**Keywords:** survival outcome, tumor size, SEER, relative death risk, parathyroid carcinoma

## Abstract

**Background:**

Parathyroid carcinoma (PC) is a rare malignancy without a commonly acknowledged prognostic assessment and treatment system. This study captures how independent prognostic factors and tumor size correlate with outcomes in patients with PC.

**Methods:**

The Surveillance, Epidemiology, and End Results database was used to perform a retrospective analysis on PC patients from 2000 to 2018. Univariate and multivariable survival analyses were performed to evaluate cancer-specific survival (CSS) and overall survival (OS), to identify independent prognostic factors in the PC patient population. A generalized additive model was applied to conduct smooth curve fitting and to examine the association between tumor size and relative risk of death.

**Results:**

A total of 590 patients were included. The 5- and 10-year OS were 80.8% and 67.1%, respectively. 5- and 10-year CSS was estimated to be 93.6% and 92.1%, respectively. The association between tumor size and relative risk of death can be generalized as a U-shaped curve. The mortality risk reaches its lowest point when tumor diameter approaches 2cm. At a tumor diameter cutoff of 3cm for CSS and 4cm for OS, there is an abrupt drop in survival rates. Multivariate Cox analysis revealed age, no surgery, and debulking surgery as consistent predictors of lower OS and CSS.

**Conclusions:**

A non-linear correlation between tumor size and death risk has been identified in patients with PC, along with an accurate size threshold at which survival rates sharply decrease. Further investigation is needed to determine if these trends are seen in other malignancies with promising prognoses.

## Introduction

Parathyroid carcinoma (PC) is a rare malignant tumor, presenting in less than 5% of all patients with primary hyperparathyroidism (PHPT) (1–4), although a higher proportion (up to 8.1%) has been reported in Chinese populations (5). Most PCs are sporadic, although they may manifest as a feature of hereditary syndromes such as multiple endocrine neoplasia (MEN) types 1 and 2A. There are no specific biochemical markers or genetic signatures for PC, and it can resemble benign parathyroid adenoma (PA) or hyperplasia (PH), which can result in the misdiagnosis of benign pathology before surgery. This casts more complexity for PC differential diagnosis and prognosis evaluation for clinical specialists.

For patients with PC, the best chance for cure is the complete excision of the tumor during the initial operation. For a surgery to be considered a success, the “gold standard” is en-bloc resection of the tumor with the ipsilateral thyroid lobe, isthmus, central neck lymph node (LN) compartment, and adjacent involved structures with gross clear margins (6, 7).

The 5-year overall survival (OS) rate is between 78 and 86%, while estimates of the 10-year rate span the range from 49 to 77% (8–10). The prognosis is based on single-center, small and retrospective cohorts, which generate conflicting outcomes and are unable to show key statistics of interest related to demographic, clinical, and treatment characteristics. As a result, the American Joint Committee on Cancer (AJCC) and similar organizations have established standalone TNM categories, but not prognostic staging groups for PC.

Some data suggests that tumor size is a better predictor of prognosis (1, 11, 12) than male gender, lymph node metastasis, age at diagnosis, or several other factors. However, it remains controversial whether tumor size can accurately predict patient outcomes, and whether there is a certain size threshold beyond which survival sharply decreases. To investigate the impact of tumor size on survival outcomes, we use a large public database to: (1) determine potential prognostic factors; (2) display overall and cancer-specific survival and verify independent predictors; and (3) explore how tumor size impacts survival outcomes in patients with PC.

## Materials and Methods

### Data Source

The study cohort was extracted from the Surveillance, Epidemiology, and End Results (SEER) program, which recodes cancer incidence and mortality data from 18 population-based cancer registries across the United States covering approximately 27.8% of the U.S. population (13). SEER registries collect data concerning patient demographics, tumor morphology, stage at diagnosis, primary tumor site, the first course of treatment, and follow-up for vital status. Our selected database is cited as: “Incidence - SEER Research Plus Data, 18 Registries, Nov 2020 Sub (2000-2018) - Linked To County Attributes - Total U.S., 1969-2019 Counties, National Cancer Institute, DCCPS, Surveillance Research Program, released April 2021, based on the November 2020 submission.” Institutional Review Board approval was not required because SEER is an open-access public database with deidentified data.

### Study Population

We included patients of all ages with confirmed PC from Incidence SEER Research Plus Database (18 Registries, 2000-2018). To acquire relevant data about patients with PC from the SEER database, we used diagnostic codes from the International Statistical Classification of Diseases and Related Health Problems 10th Revision (ICD-10). We included patients that fit the definition of a primary or metastatic malignant neoplasm affecting the parathyroid glands for ICD-10 code C75.0 (n=593). We excluded histologically distinct tumors: other non-Hodgkin lymphomas (n=1) and non-CNS paraganglioma (n=2). We extracted the following variables regarding demographic, tumor staging, and therapy fields for each record (patient): age; gender; race (white, Black, other); primary tumor site (according to tumor label, the Site Recode for Rare Tumors, and the AYA Site Recode 2020 Revision); pathological grade (2000-2017); SEER stage (2004-2017, SEER stage is defined by the SEER database referred to as the SEER stage in our article that provides information about each cancer (primary site/histology/other factors defined) schema. SEER stage includes the T, N, and M categories for each site schema, as well as the applicable Site-Specific Factors for each schema. See more information on https://seer.cancer.gov/seerstat/variables/seer/ajcc-stage/seer-combined.html); tumor size, tumor extension, and lymph node involvement (2004-2015); metastasis at diagnosis; primary site surgery; lymph node dissection (1998-2002); lymphectomy (2003); radiation; chemotherapy (yes, no/unknown); systemic treatments; cause of death; vital status (study cutoff used); and survival months.

### Statistical Analysis

In the study, the primary aim is to identify independent risk factors for overall survival (OS) and cancer-specific survival (CSS) for the entire cohort of PC patients. All anonymous data were analyzed based on demographic factors (age, gender, and race), tumor-associated parameters (pathological grading, SEER stages (14), tumor extension, lymph node involvement status, and distant metastasis) and therapy-related variables (primary site surgery, lymph node dissection, radiation, chemotherapy, and systemic treatments).We presented descriptive statistics for the entire study cohort and compared the results across SEER stages. Continuous and categorical variables were assessed with the Kruskal-Wallis test and Pearson chi-square test, respectively. Whenever the theoretical number of the counting variable is less than 10, the Fisher exact probability test will be applied.

Univariate and multivariable Cox proportional hazard models were used to estimate the effects of different covariates on both OS and CSS in study participants. OS was defined as the time from PC diagnosis to death due to any cause, and living patients were excluded from the study. CSS is represented as the time from diagnosis to death due to PC. Living patients or deaths due to causes other than PC were censored. Result estimates were presented as hazard ratios (HRs) with 95% confidence intervals (CIs). The Kaplan-Meier method was used to calculate and depict unadjusted survival curves for OS and CSS. Associations were considered statistically significant if the P-value was ≤0.05.

To further examine the association of tumor size with the survival outcomes of PC patients, we applied a generalized additive model (GAM) to conduct smooth curve fitting and to examine whether the size of the tumor is partitioned into intervals. This allowed us to determine whether the relationship with OS and CSS starts to drastically change when a tumor attains a certain size. All statistical analyses were performed with the R studio Core Team (2021, R: A language and environment for statistical computing. R Foundation for Statistical Computing, Vienna, Austria. URL https://www.R-project.org).

## Results

In total, 590 PC patients were included in the analysis. The median age of the study cohort at diagnosis was 58 years old. A balanced sex ratio was found in the PC population, with 51.5% of patients (n=304) being male and 48.5% (n=286) being female. In terms of racial composition, 75% of PC patients were white, 18% were Black, and 8% were other minority races (American Indians, Alaskan natives, Asians, and Pacific Islanders). Based on our results, the proportion of SEER stages among all PC patients was divided as follows: 44% were localized, 22% were regional, 4% were distant, and 30% were not determined. The median survival time of the entire PC cohort was 76.5 months, and the longest survival time was 226 months. **Table 1** summarizes more detailed information about patient demographics, baseline tumor characteristics, and treatments, and provides a gender comparison. Statistical tests of all variables did not show any significant gender differences.

**Table 1:** Demographic, clinical and treatment characteristics of PC patients between genders.

	Total	Gender		P
		Male	Female	
Sample size	590	304	286	
Age, median (range)	58 (14-85)	58 (23-85)	59 (14-85)	0.463
Race, N (%)				0.095
White	441 (75)	235 (77)	206 (72)	
Black	98 (17)	42 (14)	56 (20)	
Others^a^	48 (8)	24 (8)	24 (8)	
Unknown	3 (0)	3 (1)	0 (0)	
Grade, N (%)				0.508
Well differentiated; Grade I	51 (8)	27 (9)	24 (9)	
Moderately differentiated; Grade II	15 (2)	7 (2)	8 (3)	
Poorly differentiated; Grade III	3 (1)	2 (1)	1 (0)	
Undifferentiated; anaplastic; Grade IV	3 (1)	3 (1)	0 (0)	
Unknown	518 (88)	265 (87)	253 (88)	
SEER stage, N (%)				0.708
Localized	260 (44)	133 (44)	127 (44)	
Regional	128 (22)	65 (21)	63 (22)	
Distant	24 (4)	10 (3)	14 (5)	
Unknown	178 (30)	96 (32)	82 (29)	
Tumor size, mm, Mean ± SD	12.1 ± 17.9	12.08 ± 18.31	12.10 ± 17.51	0.810
Tumor extension, N (%)				0.142
Localized	282 (47.8)	153 (50.33%)	129 (45.10%)	
Regional extension	77 (13.1%)	32 (10.53%)	45 (15.73%)	
Unknown	231 (39.2.8%)	119 (39.14%)	112 (39.16%)	
Lymph nodes involvement, N (%)				0.911
No regional lymph node involved	330 (56)	170 (56)	160 (56)	
Yes	14 (2)	8 (3)	6 (2)	
Unknown	246 (42)	126 (41)	120 (42)	
Distant metastasis, N (%)				0.327
No distant metastasis	344 (58)	178 (59)	166 (58)	
Yes	6 (1)	5 (1)	1 (0)	
Unknown	240 (41)	121 (40)	119 (42)	
Primary surgery, N (%)				0.722
Parathyroidectomy	316 (54)	168 (55)	148 (52)	
En-bloc resection	233 (40)	116 (38)	117 (41)	
No surgery	25 (4)	11 (4)	14 (5)	
Debulking surgery, NOS	16 (2)	9 (3)	7 (2)	
Lymph node dissection, N (%)				0.898
No	319 (54)	167 (55)	152 (53)	
Yes	151 (26)	77 (25)	74 (26)	
Unknown	120 (20)	60 (20)	60 (21)	
Radiation, N (%)				0.071
Beam radiation	57 (10)	37 (12)	20 (7)	
Radioisotopes	6 (1)	2 (1)	4 (1)	
None/Unknown	527 (89)	265 (87)	262 (92)	
Chemotherapy, N (%)				1.000
Yes	1 (1)	1 (1)	0 (0)	
No/Unknown	589 (99)	303 (99)	286 (100)	
Systemic therapy, N (%)				0.118
No	358 (60)	191 (63)	167 (58)	
Yes	32 (6)	11 (4)	21 (7)	
Unknown	200 (34)	102 (33)	98 (35)	
Cause of death, N (%)				0.730
Alive	418 (71)	212 (70)	206 (72)	
Parathyroid carcinoma	40 (7)	20 (6)	20 (7)	
Other causes	132 (22)	72 (24)	60 (21)	
Survival months, mean ± SD	87.7 ± 63.1	88.85 ± 61.66	86.53 ± 64.68	0.502

SEER, Surveillance, Epidemiology, and End Results Program; ^a^Others, American Indian/Alaska Native, Asian/Pacific Islander. SEER stage: see Materials and Methods.

In our study population, the 5- and 10-year OS rates were 80.8% (95% CI 77.4-84.3%) and 67.1% (95% CI 62.6-71.8%), respectively. In addition, 5- and 10-year CSS rates were estimated to be 93.6% (95% CI 91.5-95.8%) and 92.1% (95% CI 89.6-94.6%), respectively. Unadjusted Kaplan-Meier survival curves are shown in **Supplementary Tables 1**, **2**. Older age (HR=1.04, 95% CI 1.03-1.05, P<0.001), Black race (vs. white, HR=2.53, 95% CI 1.72-3.74, P<0.001), no surgery (vs. local excision, HR=2.93, 95% CI 1.39-6.21, P=0.005), and use of external beam radioactive therapy (EBRT vs. no radiation, HR=1.78, 95% CI 1.06-3.00, P=0.030) were associated with a worse OS survival, after using a univariate Cox proportional hazard regression to account for demographics, tumor characteristics, and treatments. After stepwise model selection, the final multivariable Cox proportional hazards regression models showed significantly worse overall survival for older (HR=1.04, 95% CI 1.03-1.05, P<0.001) black (HR=2.34, 95% CI 1.61-3.40, P<0.001) patients who received EBRT (HR=1.69, 95% CI 1.02-2.79, P=0.04) and had no surgery (HR=3.26, 95% CI 1.72-6.18, P<0.001) or debulking surgery (HR=2.54, 95% CI 1.20-5.36, P=0.015) with no information on lymph node dissection (HR=1.76, 95% CI 1.21-2.58, P=0.003).

In R studio, there are limitations in floating-point computations as well as precision considerations. When including tumor size in millimeters as a continuous variable in a Cox regression for CSS, the system returned a computationally singular error (due to sample size difference). Therefore, we excluded tumor size from the Cox regression for CSS. Treating tumor size as a continuous variable (mm) did not have a significant impact on OS. Similar to OS, the univariate Cox model for CSS indicated that age (HR= 1.02, CI 1.00-1.05, P=0.030), African American race (vs. white, HR=2.42, CI 1.06-5.55, P=0.036) and no surgery (vs. local excision, HR=12.51, CI 3.64-43.06, P<0.001) were associated with worse survival outcomes. Moreover, unclassified SEER stage (vs. localized SEER stage, HR=16.38, 95% CI 1.04-256.86, P=0.047), regional extension (vs. extension status cannot be assessed, HR=22.91, 95% CI 1.30-403.59, P=0.032), distant metastasis (vs. no distant metastasis, HR=10.17, CI 1.13-90.99, P=0.038), debulking surgery (vs. local excision, HR=5.28, 95% CI 1.53-18.18, P=0.008), and lymph node dissection unclear (vs. lymph node dissection not performed, HR=3.69, CI 1.24-10.95, P=0.019) also negatively affected CSS.

In contrast to OS, the multivariate Cox model of CSS revealed distant SEER stage (vs. localized SEER stage, HR= 7.96, 95% CI 1.64-38.65, P=0.010) as an independent risk factor. Summaries of adjusted and unadjusted risk factors for OS and CSS can be found in **Tables 2**, **3**. Due to the low sample size, Grade: III poorly differentiated (n=3), Grade IV undifferentiated anaplastic (n=3), and chemotherapy (n=1) were not analyzed in the Cox proportional hazard regression of OS and CSS.

**Table 2:** Univariate and multivariate cox proportional hazards regression model highlighting overall survival in the parathyroid carcinoma patient population.

	Univariate		Multivariate		
	HR (95% CI)	P	HR (95% CI)		P
Age (year)	1.04 (1.03-1.05)	<0.001	1.04 (1.03-1.05)		<0.001
Gender					
Male	1 (referent)				
Female	0.91 (0.67-1.26)	0.581			
Race					
White	1 (referent)		1 (referent)		
Black	2.53 (1.72-3.74)	<0.001	2.34 (1.61-3.40)		<0.001
Others^a^	1.69 (0.95-3.01)	0.08	1.63 (0.93-2.88)		0.090
Grade					
Unknown	1 (referent)		1 (referent)		
Well differentiated; Grade I	0.88 (0.49-1.57)	0.656	0.90 (0.52-1.57)		0.715
Moderately differentiated; Grade II	0.27 (0.06-1.11)	0.069	0.29 (0.07-1.20)		0.087
SEER stage					
Localized	1 (referent)				
Unknown	3.38 (0.97-11.85)	0.057			
Reginal	0.98 (0.53-1.80)	0.942			
Distance	1.67 (0.56-4.92)	0.355			
Tumor extension					
Unknown	1 (referent)				
Localized	2.92 (0.68-12.53)	0.150			
Regional extension	3.50 (0.82-14.82)	0.090			
Tumor size (mm)	1.00 (0.98-1.01)	0.569			
Lymph nodes involvement					
No reginal lymph node involvement	1 (referent)				
Unknown	0.93 (0.36-2.39)	0.883			
Yes	2.03 (0.81-5.12)	0.133			
Distant metastasis					
No distant metastasis	1 (referent)				
Unknown	1.21 (0.56-2.62)	0.631			
Yes	3.12 (0.74-13.17)	0.121			
Primary surgery					
Parathyroidectomy	1 (referent)		1 (referent)		
En-block resection	1.14 (0.82-1.59)	0.435	1.07 (0.78-1.49)		0.665
No surgery	2.93 (1.39-6.21)	0.005	3.26 (1.72-6.18)		<0.001
Debulking surgery, NOS	2.55 (1.20-5.45)	0.016	2.54 (1.20-5.36)		0.015
Lymph node dissection					
Lymph node dissection not performed	1 (referent)		1 (referent)		
Unknown	1.59 (0.94-2.71)	0.085	1.76 (1.21-2.58)		0.003
Yes	1.29 (0.83-2.00)	0.260	1.50 (0.99-2.26)		0.054
Radiation					
None/Unknown	1 (referent)		1 (referent)		
Beam radiation	1.78 (1.06-3.00)	0.030	1.69 (1.02-2.79)		0.041
Radioisotopes	0.68 (0.09-5.28)	0.710	0.60 (0.08-4.36)		0.617
Systemic therapy					
No	1 (referent)				
Unknown	0.80 (0.51-1.24)	0.321			
Yes	0.88 (0.37-2.12)	0.784			

HR, hazard ratio; CI, confidential interval; SEER, Surveillance, Epidemiology, and End Results Program; ^a^Others, American Indian/Alaska Native, Asian/Pacific Islander. SEER stage: see Materials and Methods.

**Table 3:** Univariate and multivariate cox proportional hazards regression model highlighting cancer specific survival in the parathyroid carcinoma patient population.

	Univariate		Multivariate	
	HR (95% CI)	P	HR (95% CI)	P
Age (year)	1.02 (1.00-1.05)	0.030	1.02 (1.00-1.05)	0.039
Gender				
Male	1 (referent)			
Female	1.04 (0.54-2.02)	0.899		
Race				
White	1 (referent)			
Black	2.42 (1.06-5.55)	0.036		
Other^a^	1.81 (0.50-6.48)	0.363		
Grade				
Unknown	1 (referent)			
Well differentiated; Grade I	0.24 (0.03-1.91)	0.180		
SEER stage				
Localized	1 (referent)		1 (referent)	
Unknown	16.38 (1.04-256.86)	0.047	12.66 (0.96-166.77)	0.054
Reginal	1.39 (0.36-5.46)	0.634	2.33 (0.70-7.80)	0.170
Distance	3.14 (0.43-22.72)	0.257	7.96 (1.64-38.65)	0.010
Tumor extension				
Unknown	1 (referent)		1 (referent)	
Localized	14.00 (0.67-294.86)	0.090	7.04 (0.63-79.03)	0.114
Regional extension	22.91 (1.30-403.59)	0.032	7.72 (0.85-70.09)	0.069
Lymph nodes involvement				
No reginal lymph node involvement	1 (referent)			
Unknown	0.67 (0.09-5.11)	0.703		
Yes	2.39 (0.44-12.76)	0.309		
Distant metastasis				
No distant metastasis	1			
Unknown	2.17 (0.48-9.81)	0.316		
Yes	10.17 (1.13-90.99)	0.038		
Primary surgery				
Parathyroidectomy	1 (referent)		1 (referent)	
En-bloc resection	1.62 (0.76-3.46)	0.211	1.44 (0.69-2.99)	0.326
No surgery	12.51 (3.64-43.06)	<0.001	8.45 (2.68-26.62)	<0.001
Debulking surgery, NOS	5.28 (1.53-18.18)	0.008	3.91 (1.20-12.71)	0.024
Lymph node dissection				
Lymph node dissection not performed	1 (referent)		1 (referent)	
Unknown	3.69 (1.24-10.95)	0.019	3.53 (1.18-10.64)	0.024
Yes	0.83 (0.28-2.45)	0.740	0.98 (0.36-2.66)	0.968
Radiation				
None/Unknown	1 (referent)			
Beam radiation	2.09 (0.69-6.34)	0.195		
Systemic therapy				
No	1 (referent)		1 (referent)	
Unknown	0.41 (0.17-1.03)	0.059	0.41 (0.17-1.00)	0.050
Yes	0.70 (0.09-5.54)	0.735	0.46 (0.06-3.64)	0.460

HR, hazard ratio; CI, confidential interval; SEER, Surveillance, Epidemiology, and End Results Program; ^a^Others, American Indian/Alaska Native, Asian/Pacific Islander. SEER stage: see materials and methods.

**Figure 1:**
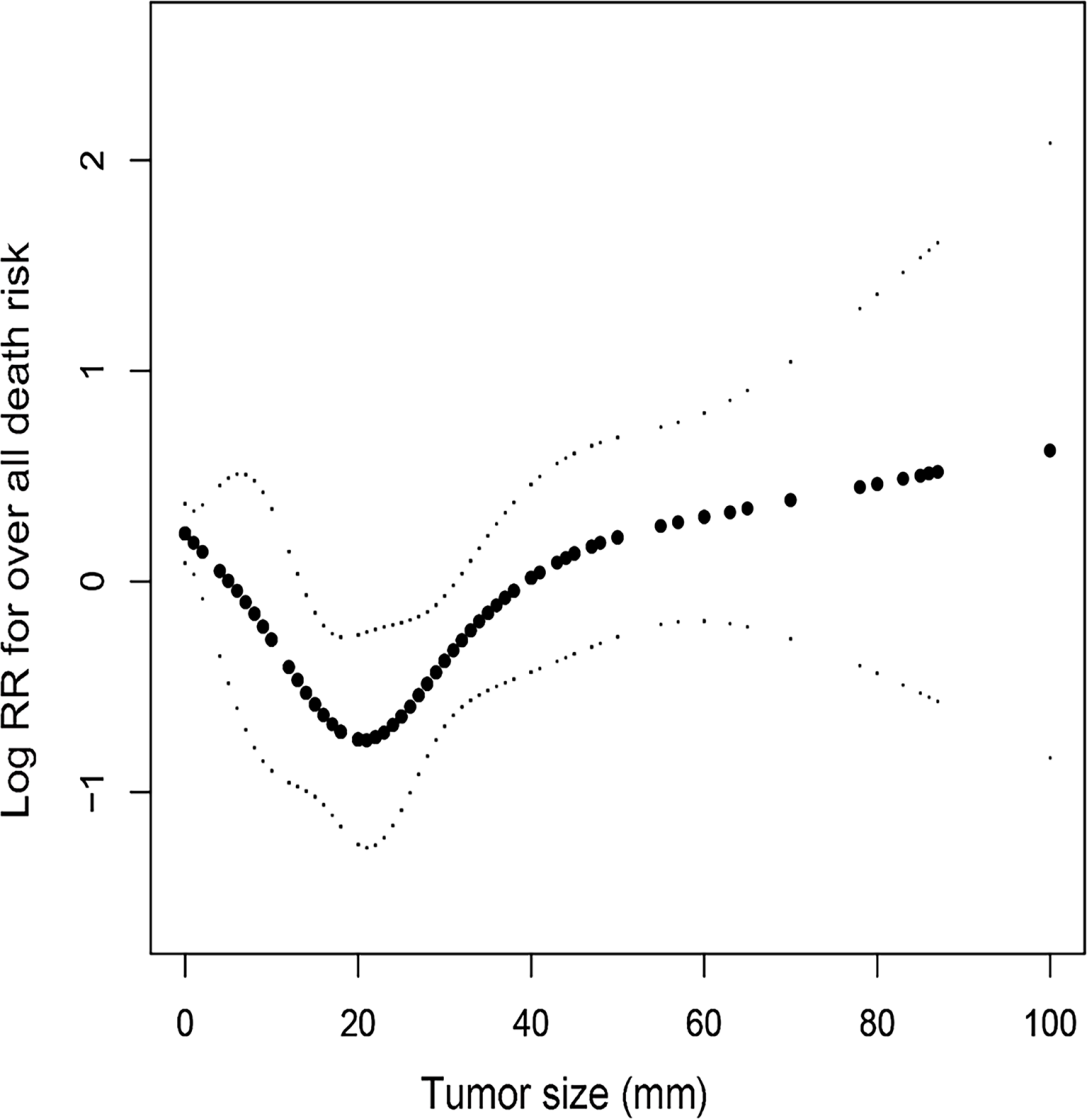
Smooth curve fitting showing the association between tumor size in millimeter and relative risk of overall death in parathyroid carcinoma patient population. The correlation between tumor size and overall death risk can be generalized as a U-shaped curve. The bottom of the curve represents parathyroid tumors with a diameter of 2 cm, and is where the risk of death reaches its lowest point.

**Figure 2:**
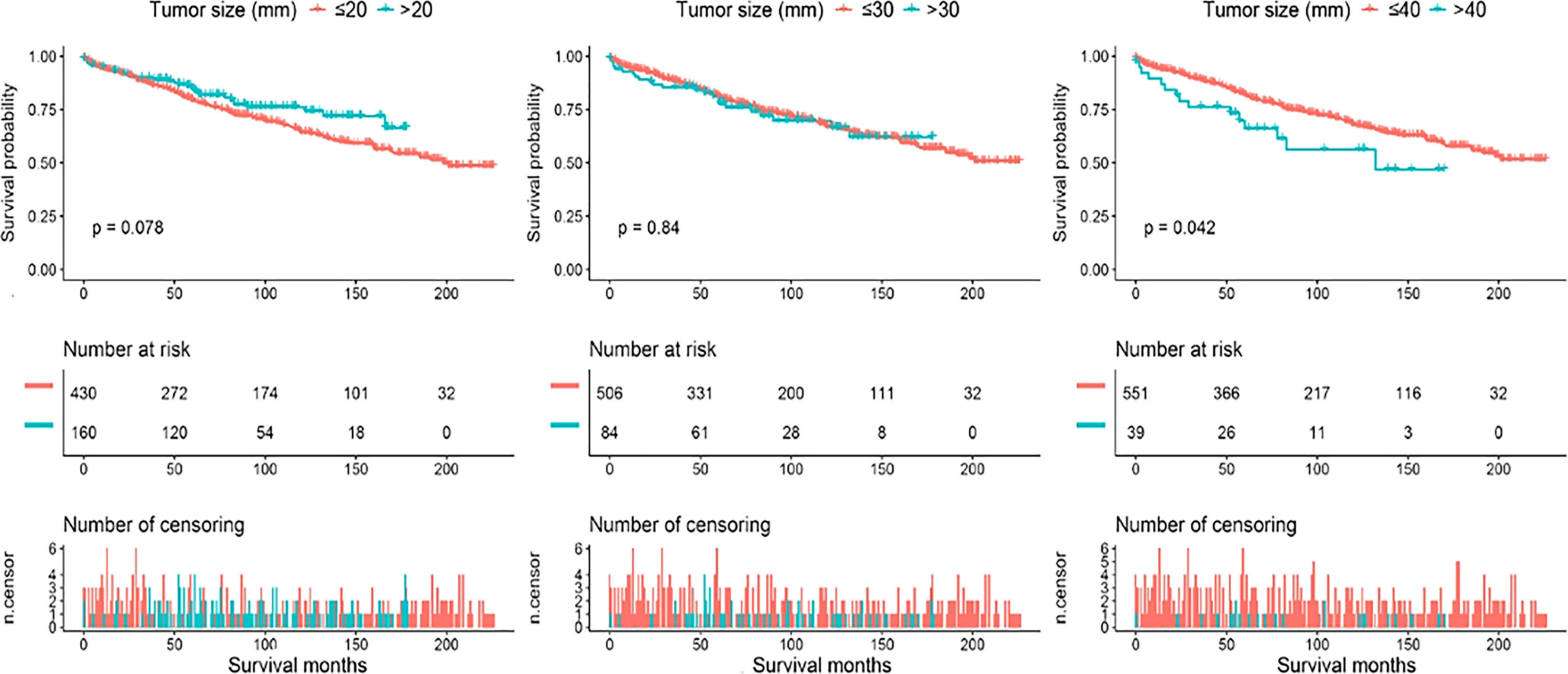
Kaplan-Meier survival curves demonstrating survival disparity of overall survival by categorizing the parathyroid carcinoma population using different tumor size cutoffs. Tumor diameter cutoffs at 2 cm, 3 cm, and 4 cm were selected as splitting points to categorize tumor size. No significant survival difference was observed between 2 cm (Log-rank test P = 0.078) and 3 cm (Log-rank test P = 0.84) cutoff groups. However, the survival disparity became statistically evident at 4 cm in tumor size (Log-rank test P = 0.042).

**Figure 3:**
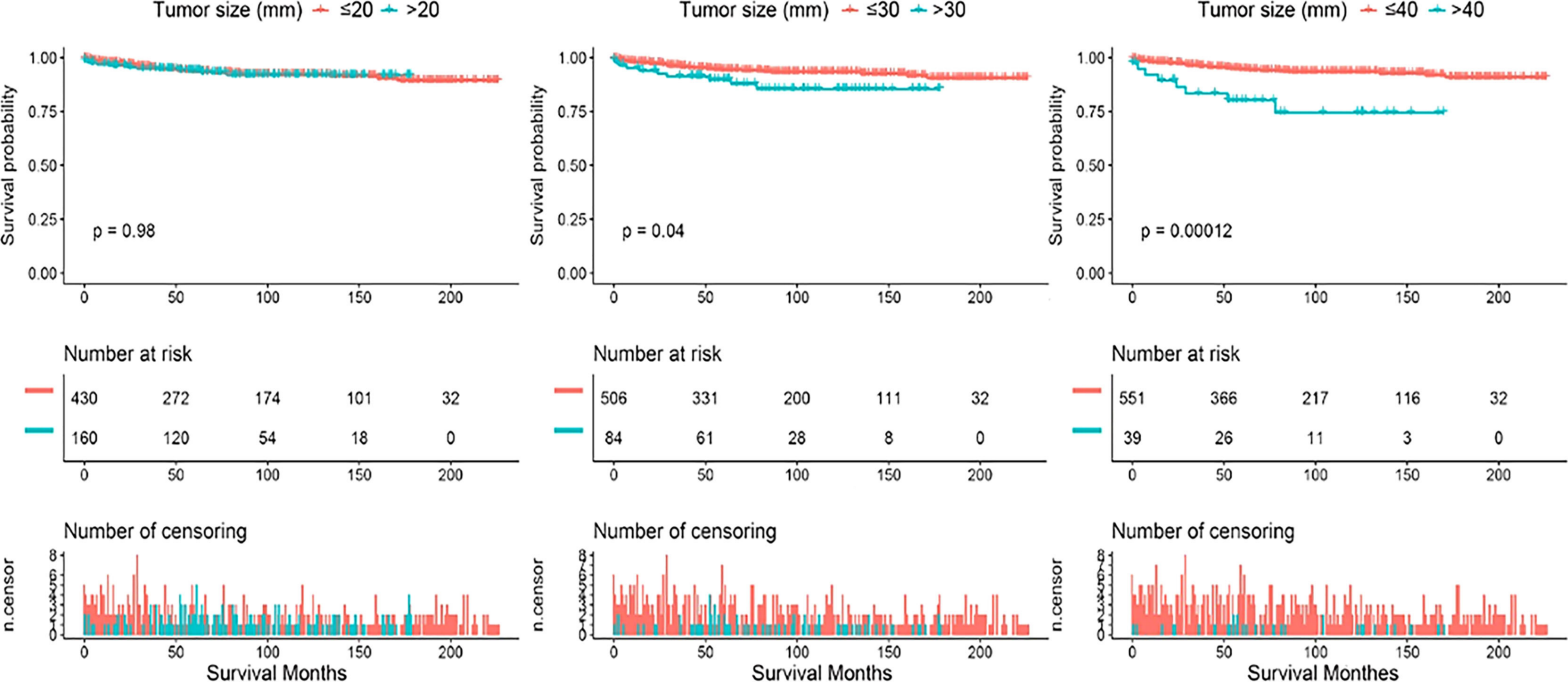
Kaplan-Meier survival curves demonstrating survival disparity of cancer-specific survival by categorizing the parathyroid carcinoma population using different tumor size cutoffs. Cancer-specific survival of PC patients was not significantly different between tumor diameter larger and small than 2 cm (Log-rank test, P=0.98) group. Yet, the cancer specific survival difference became statistically significant when dividing the PC cohort between tumor diameter cutoffs at 3 cm (Log-rank test P = 0.04) and 4 cm (Log-rank test P < 0.001).

Continuous variations in tumor size in millimeters did not yield statistically significant effects on survival, so we further plotted a smooth curve fitting (**Figure 1**). The goal was to test whether tumor size could be partitioned into categorical intervals that could distinguish survival curves. The smooth curve fitting illustrated that the association between tumor size and OS was not linear in general (Likelihood ratio test, P=0.003; Wald test, P=0.008; log rank test, P=0.006). In fact, PC patients with tumors less than approximately 4 cm in diameter had a U-shaped curve below zero of Log RR (relative risk). As tumor size increased to 2 cm, there was a decrease in the risk of death from any cause. In contrast, once tumor size exceeded 2 cm, mortality risks increased accordingly. The correlation between tumor size and cancer-specific mortality was identical to all-cause mortality (**Supplementary Figure 1**). Therefore, 2 cm, 3 cm, and 4 cm were selected as splitting points to categorize tumor size. To demonstrate survival disparities between these groups, **Figures 2** and **3** depict unadjusted Kaplan-Meier survival curves for OS and CSS, respectively. In an unadjusted Kaplan-Meier survival analysis of OS (**Figure 2**), no significant difference was observed between 2 cm (Log-rank test P=0.078) and 3 cm (Log-rank test P=0.84) groups. However, the survival difference between PC patients with tumor sizes larger and smaller than 4 cm was significant (Log-rank test P=0.042). As shown in **Figure 3**, a Kaplan-Meier survival analysis of CSS did not show a significant difference between 2 cm (Log-rank test, P=0.98) group. There was, however, a significant difference in CSS between PC patients with tumors larger and smaller than 3 cm (Log-rank test P=0.04) and 4 cm (Log-rank test P<0.001). In light of these results, we conducted a second multivariate Cox proportional hazard analysis for OS with dichotomized tumor size categories (≥4 cm and <4 cm), based on the same adjusted parameters as before. Tumors larger than 4 cm raise the risk of death by roughly 2.5 folds, when compared to those smaller than 4 cm (vs. tumor size <4 cm, HR=2.46, 95% CI 1.44-4.48, P=0.001). Other independent predictors are shown in **Supplementary Table 1**. Similarly, independent prognostic factors for CSS have been modified to use tumor size dichotomized (≥3 cm and <3 cm). In this case, tumor size, lymph node involvement, and distant metastasis become new predictors; age in years, primary surgery, and lymph node dissection remain predictors; SEER stage and systemic therapy were no longer significant predictors (**Supplementary Table 2**).

## Discussion

PCs are a group of rare malignancies that are often characterized by vastly elevated serum parathyroid hormone (PTH) and are frequently comorbid with severe hypercalcemia, also known as malignant hypercalcemia (15, 16). PC is rare among all other causes of hyperparathyroidism (parathyroid adenoma, primary parathyroid hyperplasia, parathyroid cyst, and ectopic secretion of PTH). A systematic review (17) of 22,225 cases of primary hyperparathyroidism reported that PC accounted for 0.74% of the cases, which underscores the rarity of this form of cancer. Approximately 14 years ago, a group of 224 patients with PC was identified by the SEER registry data (18) from 1988 to 2003, during which the incidence of parathyroid carcinoma increased from 3.58 to 5.73 per 10 million population.

Although PC is included in the 8th edition (released in 2017) of the cancer manual from the combined American Joint Committee on Cancer (AJCC) (19) for its TNM classification, a definitive prognostic staging system has not yet been established due to lack of large population-based evidence. To help accelerate the process of defining specific prognostic variables for PC staging, we opened a prognostic analysis primarily focused on tumor size.

The study population of 590 patients showed that PC occurred equally in female and male patients. Male gender was not an independent prognostic factor for OS (female vs. male, HR=0.91, 95% CI 0.67-1.26, P=0.581) or CSS (female vs. male, HR=1.04, 95% CI 0.54-2.02, P=0.899) in multivariate Cox analysis. This is in contrast to the conclusion of the study by Elliot et al, which showed that males had a 67% increase in the risk of death (HR=1.67, 95% CI 1.24–2.25, p = 0.0008). In fact, a recent literature review revealed conflicting results about the question of whether male patients have worse outcomes (20–22).

In our study, the 5- and 10-year OS rates were consistent with previous reports: 80.8% (95% CI 77.4-84.3%) and 67.1% (95% CI 62.6-71.8%), respectively (8–10, 12). Additionally, 5- and 10-year CSS were similar to another SEER population study: estimated at 93.6% (95% CI 91.5-95.8%) and 92.1% (95% CI 89.6-94.6%), respectively (11). This high CSS rate could be due to the fact that PC complications, such as hypercalcemia-induced renal failure, coma, and cardiac arrest, are counted as deaths from other causes instead of cancer-specific deaths in the SEER database. Therefore, the OS (80.8% at 5 years and 67.1% at 10 years) may serve as a better estimate than CSS, since SEER could potentially overestimate survival rates for this particular cancer.

Surgery is the mainstay treatment for the initial, recurrent, or metastatic onset of PCs. It is recommended that operable patients undergo en-bloc resection, which includes the ipsilateral thyroid lobe and isthmus, paratracheal alveolar and lymphatic tissue, the thymus or some of the neck muscles, and in some cases, the recurrent laryngeal nerve (23, 24). Our study found that 54% of the PC population underwent a simple parathyroidectomy and 40% underwent en-bloc surgery, whereas SEER data reported 78.6% underwent parathyroidectomy and 12.5% underwent en-bloc surgery in 2007 (18). One interpretation could be that clinicians have increasingly moved towards treating resectable PCs, and that this growing consensus is reflected in the greater proportion of en-bloc surgeries. Our multivariate OS analysis, however, indicated that en-block surgery does not significantly lower the risk of death, when compared to parathyroidectomy. It could be argued that simple parathyroidectomy is adequate for survival. In contrast, having no surgery or debulking surgery increased death risk 3.3 and 2.6 times, respectively, compared to parathyroidectomy.

Older age has previously been shown to predict poorer survival in patients with PC (18, 25). However, the age of a small cohort study of 37 patients didn’t appear to have any predictive value for survival (26). We have found that for each 1-year increase in age, there was an associated 4% increase in the risk of overall death, after adjusting for sex, race, tumor size, lymph node dissection, pathological grade, the extent of resection, and radiation (HR=1.04, 95% CI 1.03-1.05, P<0.001). To our knowledge, we are also the first group to report that each 1-year increase in age also lowers CSS for patients with PC (HR= 1.02, 95% CI 1.00-1.05, P=0.030). Similar to a recent study based on the National Cancer Database (10), our research found that the risk of death for the Black American population is 2.34 times greater than that of the white population. This may be entirely due to the relatively low socio-economic status of the Black population in the U.S.

Many studies (1, 9–12) have examined the impact of tumor size on survival outcomes. Some studies (1, 10, 18) consider tumor size as a categorical variable, with cutoff points at diameters of 2 cm, 3 cm, and 4 cm. However, no study has found that larger tumor sizes are associated with higher mortality rates. In contrast, Elliot et al. showed that tumors larger than 4 cm in diameter were associated with increased risk of death. However, tumors in the 0–1.99 cm, 2–3.99 cm, and >4 cm categories did not increase mortality risk (12). Similarly, Hsu et al. found that tumors >3 cm in diameter were associated with poorer OS (11). Small sample sizes might explain these inconsistent conclusions. Another possible interpretation is that when doing the multivariate Cox analysis for independent prognostic factors, investigators included different variables according to the focus of each study. These variables might have altered the effect of tumor size after adjustment.

To avoid statistical bias caused by differences in the variables included our multivariate analysis, we conducted smooth curve fitting and investigated the association between tumor size and overall death risk. The correlation between tumor size and OS can be generalized as a U-shaped curve (**Figure 1**). The bottom of the curve represents parathyroid tumors with a diameter of 2 cm, and is where the risk of death reaches its lowest point. The U-shaped curve probably reflects the fact that tumor size affects the likelihood of PC lesions being detected and located, or being identified and removed during surgery. As imperceptibly small tumors grow and approach 2 cm in diameter, they may become easier to diagnose thereby being resected, decreasing the risk of death. At one extreme, tumors larger than 2 cm are considered advanced T stage, with a greater chance of spreading locally or metastasizing to lymph nodes, which can significantly increase death risk. At the other extreme, PCs smaller than 2 cm are more likely to be ignored by imaging examinations, which create space and time for the carcinoma to develop and spread, thus increasing the death risk as well.

There are inherent limitations to any study relying a population-based database, and ours is no exception. The database failed to record many important variables, such as PTH and calcium levels, detailed surgical records, and disease recurrence. Those are crucial variables for prognostic evaluation in PC. Recurrence is a critical parameter for any malignant tumor. Monitoring PTH and calcium level can predict recurrence of PC. Moreover, hypercalcemia is the leading cause of death. However, the SEER database did not include records of recurrences, PTH, and serum calcium level preventing our evaluation of this point. Furthermore, clinical and oncological variables need to be accurately coded for the analysis. For instance, the third edition of the SEER coding scheme did not refer to “en-bloc resection,” and instead recoded the procedure as “radical surgery”. Consequently, the frequency of en-bloc resections was likely underestimated in our study. Moreover, pre-surgery cellular pathology was not included in SEER database and not intensively studied elsewhere, which can lead to a wrong diagnosis, so as the development of a metastatic state of PC. However, it added weight value to other prognostic factors, for instance tumor size, in the management of parathyroid carcinoma.

Despite these limitations, our study included a large cohort of PC patients for multivariate analyses of OS, CSS, and other prognostic factors.

In conclusion, PC is a rare malignancy characterized by moderate OS and outstanding CSS. Age at diagnosis, tumor size, race, and surgery are independent factors that should be considered when estimating prognosis. The association between tumor size and OS can be summarized as a U-shaped curve. When the tumor is less than 2 cm in diameter, overall mortality risk declines with tumor growth. However, overall death risk begins to rise as the tumor surpasses 2 cm, and increases sharply when the tumor exceeds 4 cm. Survival disparity of CSS becomes statistically evident as tumors grow larger than 3 cm. These findings may apply to the prognostic assessment of PC and other malignancies, and guide decision-making in regard to treatment. In our analysis, age was a constant independent predictor of bad outcomes. This observation may support a more general hypothesis that the outcome of malignancies with a promising prognosis, such as differentiated thyroid cancer, is largely influenced by a patient’s age. Future work should be extended to develop a recurrence stratification system that incorporates clinical factors, biochemical parameters (PTH, calcium level), pathological evidence (microscopic and macroscopic invasion), genetic defects (mutations and/or loss of the CDC73 tumor suppressor gene, which has been recognized to be the main genetic defects of PC) and other anatomical factors. Such a system would improve the ability to assess a patient’s chance of recurrence and survival, and lay the foundation for the development of treatment guidelines for patients with PC.

## Data Availability Statement

The original contributions presented in the study are included in the article/**Supplementary Material**. Further inquiries can be directed to the corresponding author.

## Author Contributions

KZ and Y-WC contributed to concept and design of the study, acquisition, analysis, and interpretation of data, writing–initial draft, critical revision of the article for important intellectual content, had full access to all data, and had final responsibility for the decision to submit the article for publication. AS, XW, WZ, and LH contributed to data acquisition, analysis, and interpretation of data, critical revision of the article for important intellectual content. TW, ZL, and JZ contributed to concept and design of the study, acquisition and analysis of data.

## Conflict of Interest

The authors declare that the research was conducted in the absence of any commercial or financial relationships that could be construed as a potential conflict of interest.

## Publisher’s Note

All claims expressed in this article are solely those of the authors and do not necessarily represent those of their affiliated organizations, or those of the publisher, the editors and the reviewers. Any product that may be evaluated in this article, or claim that may be made by its manufacturer, is not guaranteed or endorsed by the publisher.

## Supplementary Material

Supplementary Figure 1 Kaplan-Meier survival curve demonstrating overall survival.

Supplementary Figure 2 Kaplan-Meier survival curve demonstrating cancer-specific survival.

Supplementary Figure 3 Smooth curve fitting showing the association between tumor size in millimeter and relative risk of cancer-specific death in parathyroid carcinoma patient population.
