# Supplementary figures and images for: Non-Linear Correlation Between Tumor Size and Survival Outcomes for Parathyroid Carcinoma: A SEER Population-Based Cohort Study
Source: Front Endocrinol (Lausanne). 2022 Jul 1;13:882579. doi: 10.3389/fendo.2022.882579 (PMC9285012; doi:10.3389/fendo.2022.882579)

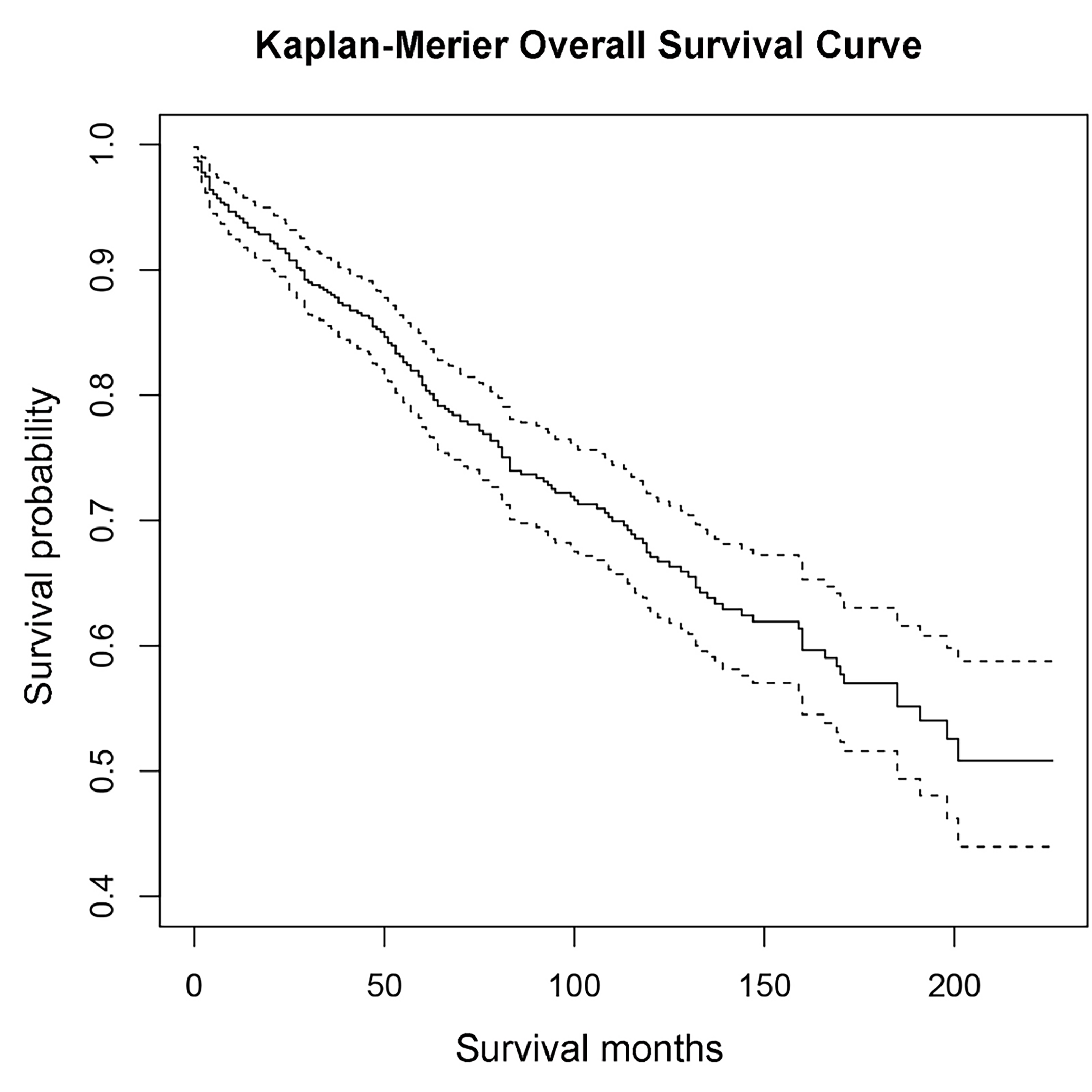

Supplement: Supplementary Figure 1 — Kaplan-Meier survival curve demonstrating overall survival. [file Image_1.tif]

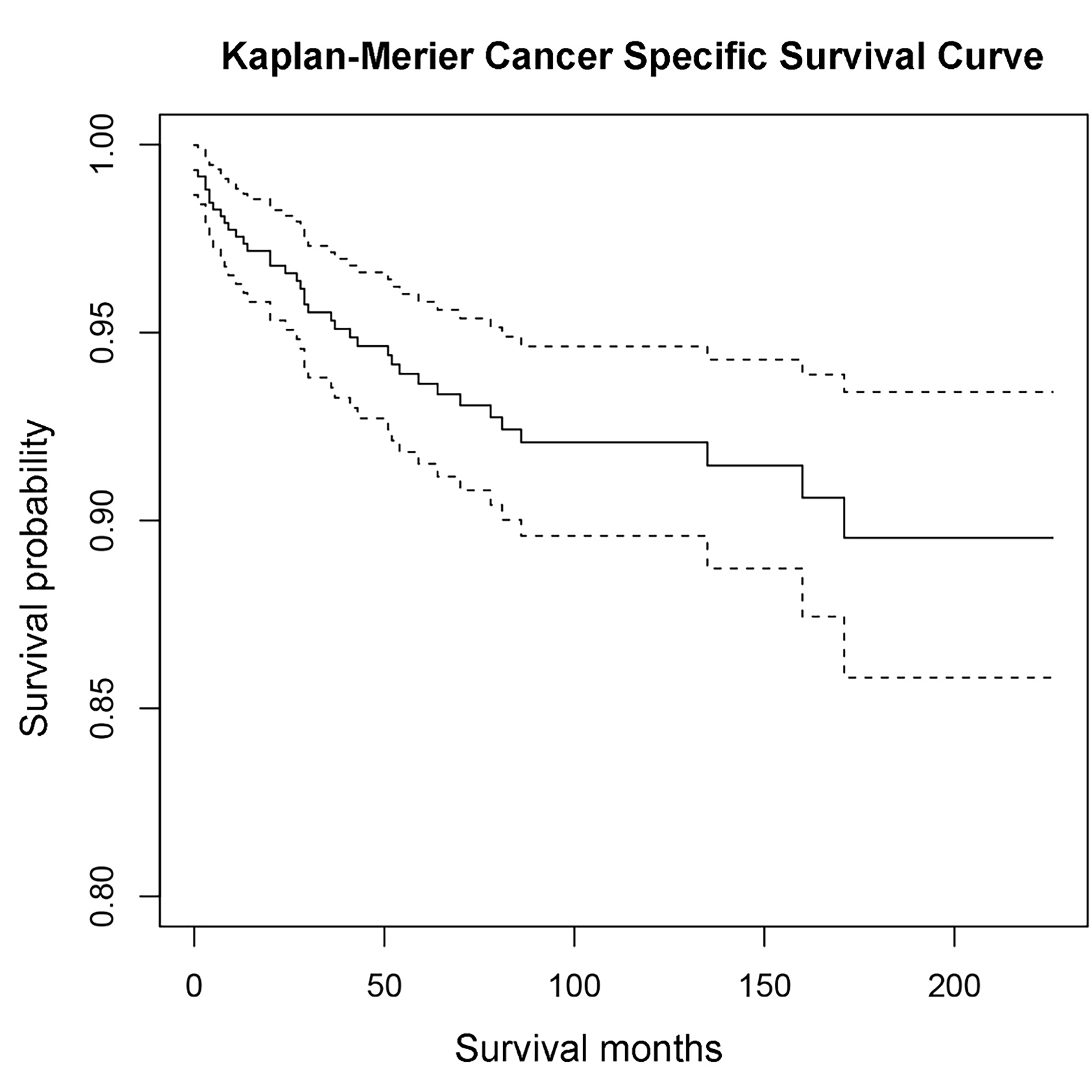

Supplement: Supplementary Figure 2 — Kaplan-Meier survival curve demonstrating cancer-specific survival. [file Image_2.tif]

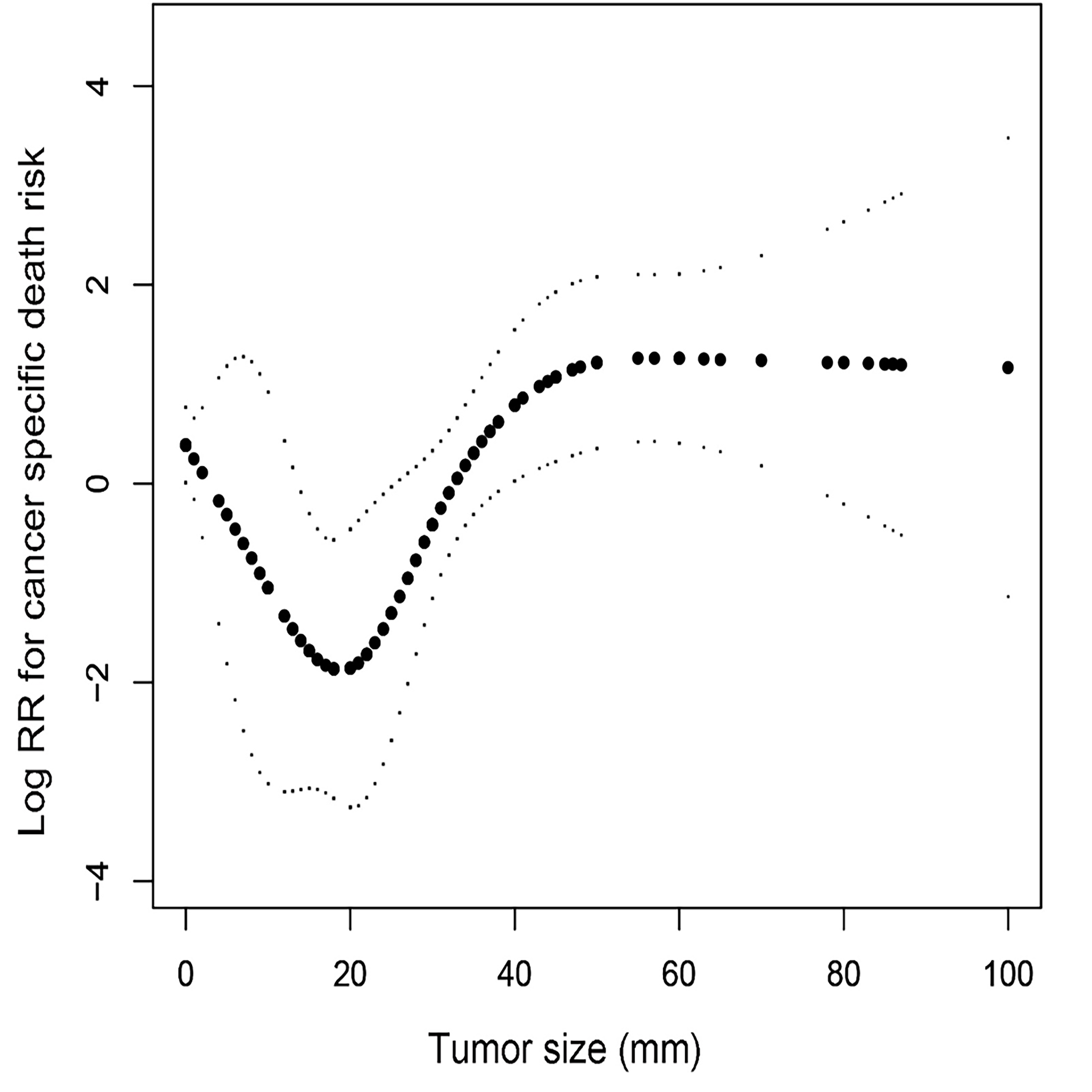

Supplement: Supplementary Figure 3 — Smooth curve fitting showing the association between tumor size in millimeter and relative risk of cancer-specific death in parathyroid carcinoma patient population. [file Image_3.tif]
