# Supplementary material for: Non-Linear Correlation Between Tumor Size and Survival Outcomes for Parathyroid Carcinoma: A SEER Population-Based Cohort Study
Source: Front Endocrinol (Lausanne). 2022 Jul 1;13:882579. doi: 10.3389/fendo.2022.882579 (PMC9285012; doi:10.3389/fendo.2022.882579)
Supplement: Supplementary file 4 [file Table_1.pdf]

Supplementary Table 1. Univariate and Multivariate Cox Proportional Hazards Regression Model Highlighting Overall Survival in the Parathyroid Carcinoma Patient Population with dichotomous tumor size.

		Univariate		Multivariate	
		HR (95% CI)	P	HR (95% CI)	P
Age (year)		1.04 (1.03-1.05)	<0.001	1.04 (1.03-1.05)	0.000
Gender					
	Male	1 (referent)			
	Female	0.90 (0.65-1.23)	0.508		
Race					
	White	1 (referent)		1 (referent)	
	Black	2.38 (1.61-3.51)	<0.001	2.16 (1.48-3.15)	0.000
	Other	1.63 (0.92-2.90)	0.096	1.61 (0.91-2.83)	0.102
Grade					
	Unknown	1 (referent)		1 (referent)	
	Well differentiated; Grade I	0.90 (0.50-1.61)	0.726	1.01 (0.58-1.77)	0.961
	Moderately differentiated; Grade II	0.27 (0.07-1.13)	0.074	0.31 (0.08-1.26)	0.101
SEER stage					
	Localized	1 (referent)		1 (referent)	
	Unknown	3.45 (0.98-12.15)	0.054	1.47 (0.87-2.47)	0.148
	Reginal	0.95 (0.52-1.73)	0.855	1.22 (0.77-1.91)	0.399
	Distance	1.60 (0.54-4.74)	0.396	2.36 (1.20-4.65)	0.013
Tumor extension					
	Unknown	1 (referent)			
	Localized	2.73 (0.63-11.72)	0.178		
	Regional extension	3.57 (0.84-15.26)	0.086		
Tumor size		1.00 (0.98-1.01)	0.569		
	<4cm	1 (referent)		1 (referent)	
	≥4cm	2.46 (1.37-4.40)	0.003	2.54 (1.44-4.48)	0.001
Lymph nodes involvement					
	No reginal lymph node involvement	1 (referent)			
	Unknown	1.02 (0.39-2.67)	0.964		
	Yes	1.99 (0.79-5.04)	0.145		
Distant metastasis					
	No distant metastasis	1 (referent)			
	Unknown	1.24 (0.57-2.72)	0.590		
	Yes	3.30 (0.77-14.18)	0.108		

Primary surgery				
Parathyroidectomy	1 (referent)		1 (referent)	
En-bloc resection	1.10 (0.79-1.53)	0.592	1.04 (0.75-1.44)	0.827
No surgery	3.01 (1.42-6.38)	0.004	2.39 (1.17-4.88)	0.017
Debulking surgery, NOS	2.40 (1.13-5.12)	0.023	2.31 (1.09-4.87)	0.029
Lymph node dissection				
Lymph node dissection not performed	1 (referent)		1 (referent)	
Unknown	1.64 (0.96-2.80)	0.069	1.55 (0.92-2.63)	0.102
Yes	1.34 (0.86-2.08)	0.196	1.51 (0.99-2.29)	0.055
Radiation				
None/Unknown	1 (referent)			
Beam radiation	1.62 (0.97-2.71)	0.066		
Radioisotopes	0.72 (0.09-5.50)	0.750		
Systemic therapy				
No	1 (referent)			
Unknown	0.76 (0.49-1.19)	0.232		
Yes	0.95 (0.40-2.29)	0.913		

Abbreviations: HR, hazard ratio; CI, confidential interval; SEER, Surveillance, Epidemiology, and End Results Program; [a]Others, American Indian/Alaska Native, Asian/Pacific Islander.
