# Supplementary material for: Non-Linear Correlation Between Tumor Size and Survival Outcomes for Parathyroid Carcinoma: A SEER Population-Based Cohort Study
Source: Front Endocrinol (Lausanne). 2022 Jul 1;13:882579. doi: 10.3389/fendo.2022.882579 (PMC9285012; doi:10.3389/fendo.2022.882579)
Supplement: Supplementary file 5 [file Table_2.pdf]

Supplementary Table 2. Univariate and Multivariate Cox Proportional Hazards Regression Model Highlighting Cancer Specific Survival in the Parathyroid Carcinoma Patient Population with Dichotomous tumor size.

		Univariate		Multivariate	
		HR (95% CI)	P	HR (95% CI)	P
Age (year)		1.03 (1.01-1.06)	0.014	1.03 (1.00-1.05)	0.028
Gender					
	Male	1 (referent)			
	Female	1.14 (0.58-2.24)	0.711		
Race					
	White	1 (referent)			
	Black	1.88 (0.81-4.36)	0.142		
	Other	1.66 (0.47-5.83)	0.427		
Grade					
	Unknown	1 (referent)			
	Well differentiated; Grade I	0.30 (0.04-2.25)	0.240		
SEER stage					
	Localized	1 (referent)			
	Unknown	3.99 (0.85-18.71)	0.080		
	Reginal	2.14 (0.77-5.90)	0.143		
	Distance	3.26 (0.68-15.57)	0.138		
Tumor size		1.00 (0.98-1.01)	0.569		
	<3cm	1 (referent)		1 (referent)	
	≥3cm	3.90 (1.53-9.92)	0.004	4.49 (1.81-11.17)	0.001
Lymph nodes involvement					
	No reginal lymph node involvement	1 (referent)		1 (referent)	
	Unknown	0.21 (0.04-1.25)	0.087	0.19 (0.04-0.97)	0.045
	Yes	1.46 (0.22-9.63)	0.692	2.22 (0.34-14.77)	0.408
Distant metastasis					
	No distant metastasis	1 (referent)		1 (referent)	
	Unknown	2.90 (0.56-15.09)	0.207	4.96 (1.09-22.48)	0.038
	Yes	7.00 (0.77-63.77)	0.084	9.55 (1.66-54.97)	0.012
Primary surgery					
	Parathyroidectomy	1 (referent)		1 (referent)	
	En-bloc resection	1.46 (0.69-3.10)	0.327	1.37 (0.66-2.86)	0.403
	No surgery	9.01 (2.64-30.75)	0.000	11.89 (3.80-37.21)	0.000
	Debulking surgery, NOS	3.97 (1.15-13.73)	0.029	4.93 (1.56-15.61)	0.007
Lymph node dissection					
	Lymph node dissection not performed	1 (referent)		1 (referent)	
	Unknown	3.61 (1.19-10.95)	0.023	3.69 (1.33-10.23)	0.012
	Yes	0.82 (0.27-2.48)	0.725	0.74 (0.25-2.20)	0.592
Radiation					
	None/Unknown	1 (referent)			
	Beam radiation	1.45 (0.47-4.42)	0.516		
Systemic therapy					
	No	1 (referent)			
	Unknown	0.48 (0.19-1.22)	0.122		
	Yes	0.71 (0.09-5.57)	0.742		

Abbreviations: HR, hazard ratio; CI, confidential interval; SEER, Surveillance, Epidemiology, and End Results Program; [a]Others, American Indian/Alaska Native, Asian/Pacific Islander.
